# Prevalence and association of Molar Incisor Hypomineralization (MIH) in patients presenting dental fluorosis: a systematic review and meta-analysis

**DOI:** 10.1186/s12903-026-08052-9

**Published:** 2026-03-07

**Authors:** Inas Bahloul, Ghita Elbasraoui, Sonia Ghoul

**Affiliations:** International Faculty of Dental Medicine, College of Health Sciences, Health Science Research Center (CReSS), International University of Rabat, Rabat, Morocco

**Keywords:** Molar Hypomineralization, Dental Fluorosis, Developmental Defects of Enamel, Fluorides, Systematic Review, Meta-analysis

## Abstract

**Background:**

Molar Incisor Hypomineralization (MIH) and Dental Fluorosis (DF) are two developmental enamel defects with overlapping clinical features. From an epidemiological perspective, studying the co-occurrence of MIH and DF provides a more comprehensive view of enamel health, helping to avoid underestimation of their true prevalence and to identify shared environmental risk patterns. Despite their frequent co-occurrence, evidence regarding a potential relationship between MIH and DF remains unclear. This systematic review and meta-analysis aimed to assess the prevalence of MIH among children with DF and to investigate the possible association between these two conditions.

**Methods:**

Following PRISMA guidelines, a systematic search was conducted in PubMed, Scopus, Web of Science, and the Cochrane Library. Cross-sectional studies reporting the prevalence of MIH in populations with DF were included. Two reviewers performed study selection, data extraction, and quality assessment independently. A meta-analysis with a random-effects model was conducted in RevMan.

**Results:**

Five cross-sectional studies, including 3,071 children aged 8–16 years, met the inclusion criteria. Across the included studies, the prevalence of MIH and DF was 21% and 51%, respectively. The prevalence of co-occurrence ranged from 3.7% to 26%. A statistically significant association between MIH and DF was observed (χ² = 10.1, *p* = 0.002; OR = 1.36, 95% CI: 1.12–1.65). However, the meta-analysis based on four studies (*n* = 1,503) did not confirm this association (OR = 1.11, 95% CI: 0.86–1.42, *p* = 0.43), with moderate heterogeneity (I² = 56%). Most MIH cases were of mild severity (55.1%).

**Conclusions:**

While descriptive analyses suggested a potential relationship between MIH and DF, meta-analytic results did not support a significant association. These findings highlight the need for further standardized diagnostic criteria and well-calibrated epidemiological studies to clarify whether environmental fluoride exposure influences MIH occurrence.

**Trial registration:**

This systematic review was registered in the PROSPERO database (ID: CRD42024628754).

**Supplementary Information:**

The online version contains supplementary material available at 10.1186/s12903-026-08052-9.

## Introduction

 Developmental enamel defects (DDEs) are a group of conditions in which dental enamel is altered during its formation process, known as amelogenesis. These defects are permanent because enamel, as the outer layer of the tooth crown, cannot be regenerated. Therefore, DDEs represent a frequent challenge in pediatric dentistry. The etiology of these DDEs is multifactorial and includes genetic, epigenetic, and environmental factors during tooth development. Exposure to these factors, along with their timing and duration, largely determines the specific type and extent of the defect [1]. DDEs are typically classified into two main types: quantitative defects, such as enamel hypoplasia, and qualitative defects, such as enamel hypomineralization. Hypoplasia is characterized by a reduced amount of enamel, often presenting pits, grooves, or a partial absence of the enamel layer. In contrast, hypomineralization refers to insufficient mineralization of the enamel, either localized or generalized, leading to discoloration and increased porosity of the tissue [2]. Depending on severity, DDEs may affect one or several teeth and are commonly associated with clinical concerns such as poor aesthetics, increased dental sensitivity, changes in occlusion, and a greater risk of developing dental caries [3, 4]. Dental Fluorosis (DF) is an enamel defect that results from chronic and excessive intake of fluoride during enamel formation [5]. This condition is characterized by subsurface hypomineralization and increased enamel porosity beneath a relatively well-mineralized surface [6]. This hypomineralization is closely linked to alterations in enamel structure and changes in tooth color. Thus, teeth may appear white-creamy when they are less porous, or brown when they are more porous. Clinically, DF lesions are symmetric and painless with a spectrum of severities varying from white opacities to more pronounced structural damage, depending on the dose and duration of fluoride exposure [7]. To determine the severity of DF, several clinical indices might be used, including the Dean’s index, which classifies DF into six categories [8], the Thylstrup and Fejerskov index, where scores range from 0 to 9, and the Tooth Surface Index of Fluorosis (TSIF), which evaluates individual tooth surfaces from 0 to 7 [9].

Additionally, Molar Incisor Hypomineralization (MIH) is a qualitative enamel defect affecting one to four first permanent molars (FPMs) and may also affect permanent incisors. MIH is characterized by demarcated opacities of varying colors, including white, cream, yellow, and brown. As the condition progresses, these opacities may lead to post-eruptive enamel breakdown. However, not all MIH lesions exhibit breakdown. Therefore, post-eruptive enamel breakdown is considered a possible consequence rather than a defining diagnostic criterion. The etiology of MIH remains unclear and is considered multifactorial, involving genetic, environmental, and medical factors that may occur during early childhood [10]. Clinically, MIH lesions are asymmetric and often associated with hypersensitivity, which may progress to pain in more severe cases. The European Academy of Pediatric Dentistry (EAPD) criteria remain the most widely adopted tool for the diagnosis of MIH, followed by the DDE index and the classification proposed by Ghanim et al. [11, 12]. In addition to diagnostic tools, indices such as the MIH severity grading scales and the MIH treatment need index (MIH-TNI) have been developed to assess lesion severity and guide the clinical management of MIH [13].

Despite having seemingly distinct etiologies, both MIH and DF present qualitative enamel defects and increasingly overlap in their presentations, manifesting clinical similarities and leading to diagnostic challenges [14]. The role of fluoride during dental enamel development remains questionable, as it may act either as a protective factor or as a potential aggravating factor of DDEs. The variability in the clinical presentation of DF within a population submitted to the same fluoride exposure suggests that fluoride alone may not fully explain the observed DF. Moreover, fluoride may interact with various systemic and environmental factors, suggesting an influence on enamel formation in various ways [15]. Overlapping risk zones where children might be exposed to both excessive fluoride and systemic and environmental factors may suggest potential hypotheses concerning whether co-exposure leads to interactions. However, the relationship between MIH and DF remains unclear. In fact, some studies conducted in fluoridated populations have reported a lower prevalence and severity of MIH, particularly in cases of high-severity fluorosis [16], whereas others have suggested that fluoride exposure may contribute to the development or worsening of MIH, depending on the concentration and timing of exposure [17]. Studying the co-occurrence of MIH and DF is clinically relevant and scientifically important. This study aims to fill a gap in current epidemiological evidence by assessing the prevalence and potential association between MIH and DF. The main objective of this systematic review and meta-analysis was to estimate the prevalence of MIH among children with DF and to evaluate the potential relationship between these two enamel defects. Investigating their co-occurrence is relevant because both conditions result from disturbances in enamel mineralization and may share biological pathways, genetic susceptibilities, or environmental triggers. Although DF is associated with chronic fluoride exposure, MIH is linked to disturbances occurring during the late maturation stage of enamel development. Their coexistence in the same patient could reveal overlapping etiological mechanisms, particularly in areas with variable fluoride exposure or additional environmental stressors. Clinically, understanding mixed phenotypes can enhance diagnosis, prevention, and decision-making. Epidemiologically, it may refine prevalence estimates and support integrated models linking biological, environmental, and oral factors in enamel development.

To our knowledge, no systematic review has quantitatively synthesized epidemiological evidence on the co-occurrence of MIH and DF. Clarifying this relationship is important for improving diagnostic differentiation and for better interpreting variations in reported DDEs prevalence in populations where fluoride exposure is common.

## Materials and methods

### Review design

This systematic review was registered in the PROSPERO database (ID: CRD42024628754) and was conducted following the PRISMA (Preferred Reporting Items for Systematic Reviews and Meta-Analyses) guidelines.

### Focused question and eligibility criteria

Based on the review’s aim, the research question was defined as follows: “What is the prevalence of MIH in individuals diagnosed with DF, and is there a significant association between these two conditions?”.

Eligibility criteria were predefined using the PECOS model (population, exposure, comparator, outcome, study design) to ensure reproducibility and methodological rigor (Table 1).

**Table 1:** PECOS schema: Population (P), Exposure (E), Comparison (C), Outcomes (O), and Study Design (S)

*P*	Participants: children, adolescents, and elderly patients diagnosed with both MIH (mild, moderate, and severe) and DF (any severity)
E	Exposure: Patients living in endemic fluoride areas
C	No comparison group specified
O	Primary outcome: Prevalence of MIH in patients with DF Secondary outcome: Association between MIH and DF
S	Study types: observational studies including cohort, case-control, and cross-sectional studies

### Inclusion criteria

Studies were included if they met the following conditions: (i) cross-sectional observational studies involving participants clinically diagnosed with DF, and (ii) studies that assessed the presence of MIH, including both mild, moderate, and severe forms. Although the initial protocol aimed to include all observational designs, only cross-sectional studies met the eligibility criteria after full-text screening.

### Exclusion criteria

Studies were excluded if they: (i) did not assess the prevalence of MIH among individuals with DF, (ii) did not report data on the diagnosis or prevalence of DF, or (iii) did not examine or report any statistical associations between MIH and DF. These criteria were used to identify the most relevant studies to the review’s objectives.

### Study identification and selection

Studies were identified using search strategies that were specifically designed for each of the following databases: PubMed, Scopus, and Web of Science (Appendix 1). A manual search of the reference lists from relevant articles was also carried out. The search was conducted in January 2025. The search strategy involved two concepts related to MIH and DF linked by the Boolean operator AND. Each concept was developed with synonyms as follows: Concept 1: (“Molar Hypomineralization”) OR (“Hypomineralization Molar”) OR (“Molar Hypomineralizations”) OR (“Molar Incisor Hypomineralization”) OR (“Hypomineralization, Molar Incisor”) OR (“Molar Incisor Hypomineralizations”) OR (“Incisor Molar Hypomineralization”) OR (“Hypomineralization Incisor Molar”) OR (“Incisor Molar Hypomineralizations”) OR (“Molar Hypomineralization Incisor”) OR (“Incisor Hypomineralization”) OR (“Hypomineralization, Incisor”) OR (“Incisor Hypomineralizations”) OR (“MIH”). Concept 2: (“Fluorosis Dental”) OR (Fluoride) OR (“Dental Fluorosis”) OR (“Dental Fluoroses”) OR (“Fluoroses Dental”) OR (“Mottled Teeth”) OR (“Teeth Mottled”) OR (“Mottled Enamel”) OR (“Enamel Mottled”) OR (“Mottled Enamels”) OR (“Fluorides”). Study selection was managed using the Rayyan AI tool, which facilitated the removal of duplicates and the screening of titles and abstracts. Full-text articles were then reviewed to assess eligibility. This process was performed independently by two reviewers, and any disagreements were resolved through discussion and consensus.

### Data extraction process

Data extraction was carried out independently by two reviewers using a Cochrane-based grid specifically adapted for this review. The following information was collected from each study: (i) Citation details such as first author, year, journal, title, country, and corresponding author contact, (ii) study characteristics such as objective, design, setting, location and recruitment methods, inclusion/exclusion criteria, and any declared funding or conflicts of interest, (iii) population data such as sample size, age, gender distribution, fluoride concentration in drinking water, and whether the area was considered endemic or not, and (iv) Outcome data such as diagnostic criteria for MIH and DF, examiner calibration and inter-examiner agreement (Cohen’s kappa, κ), stage of dentition (early mixed, late mixed, permanent), severity of MIH (mild, moderate, severe) and affected teeth. MIH severity was recorded according to the classification systems used in each included study. MIH severity generally reflects the extent of enamel involvement. Mild cases typically present demarcated opacities without post-eruptive enamel breakdown, moderate cases may include limited enamel breakdown and/or hypersensitivity, and severe cases are characterized by extensive breakdown, atypical restorations, or marked hypersensitivity affecting function. For studies with a control group, the same variables were extracted. Disagreements between reviewers were resolved through discussion or, if necessary, with a third reviewer.

### Risk of bias assessment

The risk of bias and methodological quality of the included studies was independently evaluated using an adapted framework based on the Joanna Briggs Institute (JBI) Critical Appraisal Checklist for Analytical Cross-Sectional Studies. To capture the specific aspects of prevalence and fluoride exposure studies, the tool was expanded to include 12 domains: clarity of inclusion criteria, population representativeness, validity of fluoride exposure assessment, diagnostic criteria for MIH and DF, identification of confounders, examiner calibration (κ), response rate, missing data reporting, and appropriateness of statistical analysis. Each domain was rated as low risk (+), some concerns (–), or high risk (×). Any disagreements between reviewers were resolved through discussion until consensus was reached.

### Data analyses

Statistical analyses were conducted using Jamovi (version 2.3.28) and Review Manager (RevMan, version 5.4). Descriptive and inferential analyses were first performed in Jamovi. Contingency tables were used to assess the associations between MIH and DF. ORs with 95% CIs were calculated, and significance was tested using chi-square tests and Fisher’s exact tests. For the meta-analysis, RevMan was used to pool study-specific ORs under a random-effects Mantel–Haenszel model to account for between-study variability. Heterogeneity was quantified with I² and τ² and explored with leave-one-out sensitivity analyses. One study was identified as an outlier that disproportionately increased heterogeneity. The outlier was retained in the qualitative synthesis. Forest plots were generated to display individual and pooled effects. Publication bias was explored by visual inspection of a funnel plot.

## Results

### Selection and characteristics of the papers

**Fig. 1:**
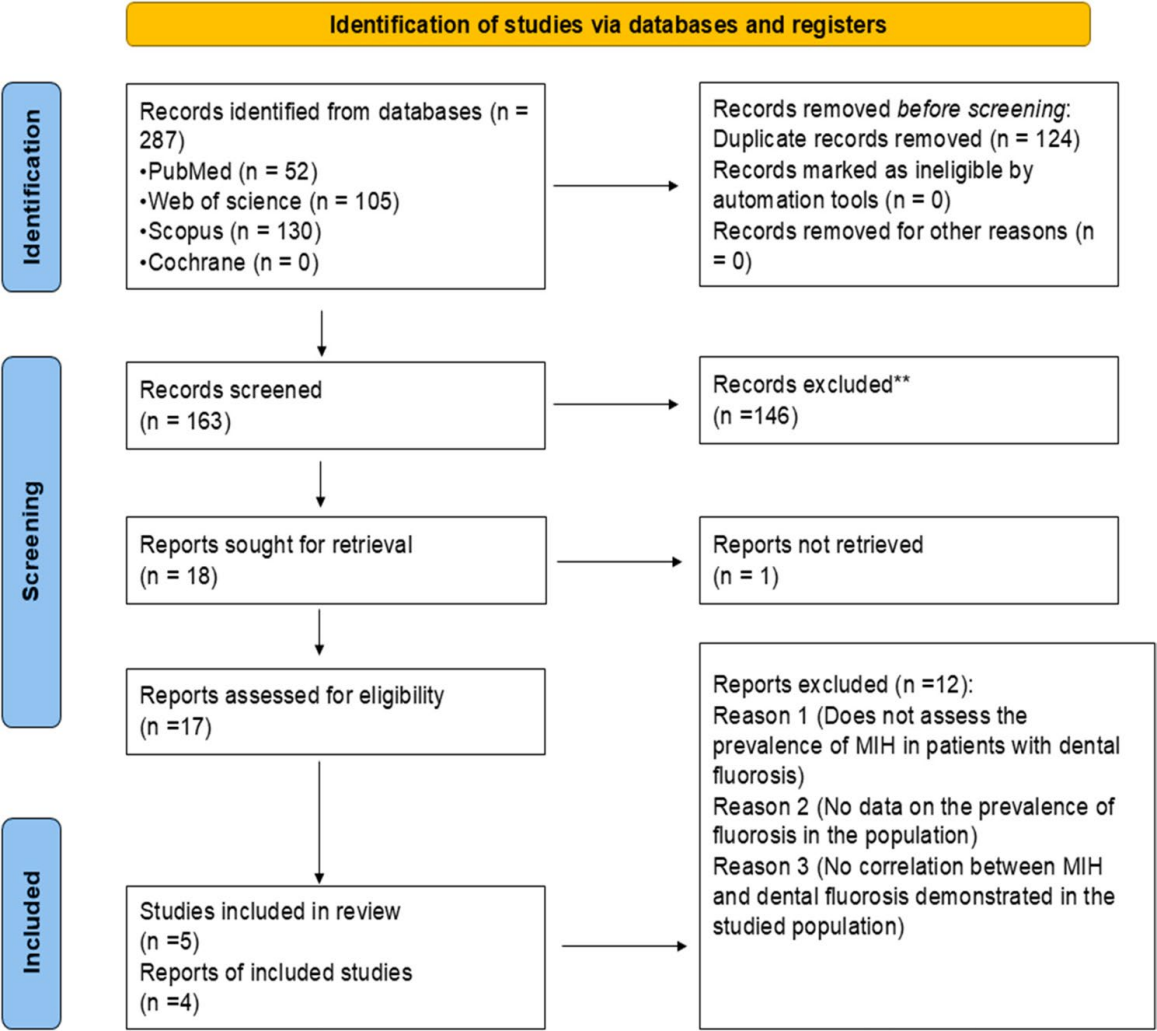
PRISMA flow diagram for study selection

A total of 287 articles were initially identified through electronic and manual literature searches. After removing duplicates (*n* = 124), 163 articles underwent further screening based on titles and abstracts, from which 146 were excluded due to irrelevance. One article was not retrieved. A total of 17 articles were assessed for full-text eligibility. Twelve studies were excluded according to the inclusion and exclusion criteria. Finally, five cross-sectional studies were included in this systematic review. The PRISMA flow chart presenting the screening process for this study is illustrated in Fig. 1 (Fig. 1).

### Risk of bias assessment

**Fig. 2:**
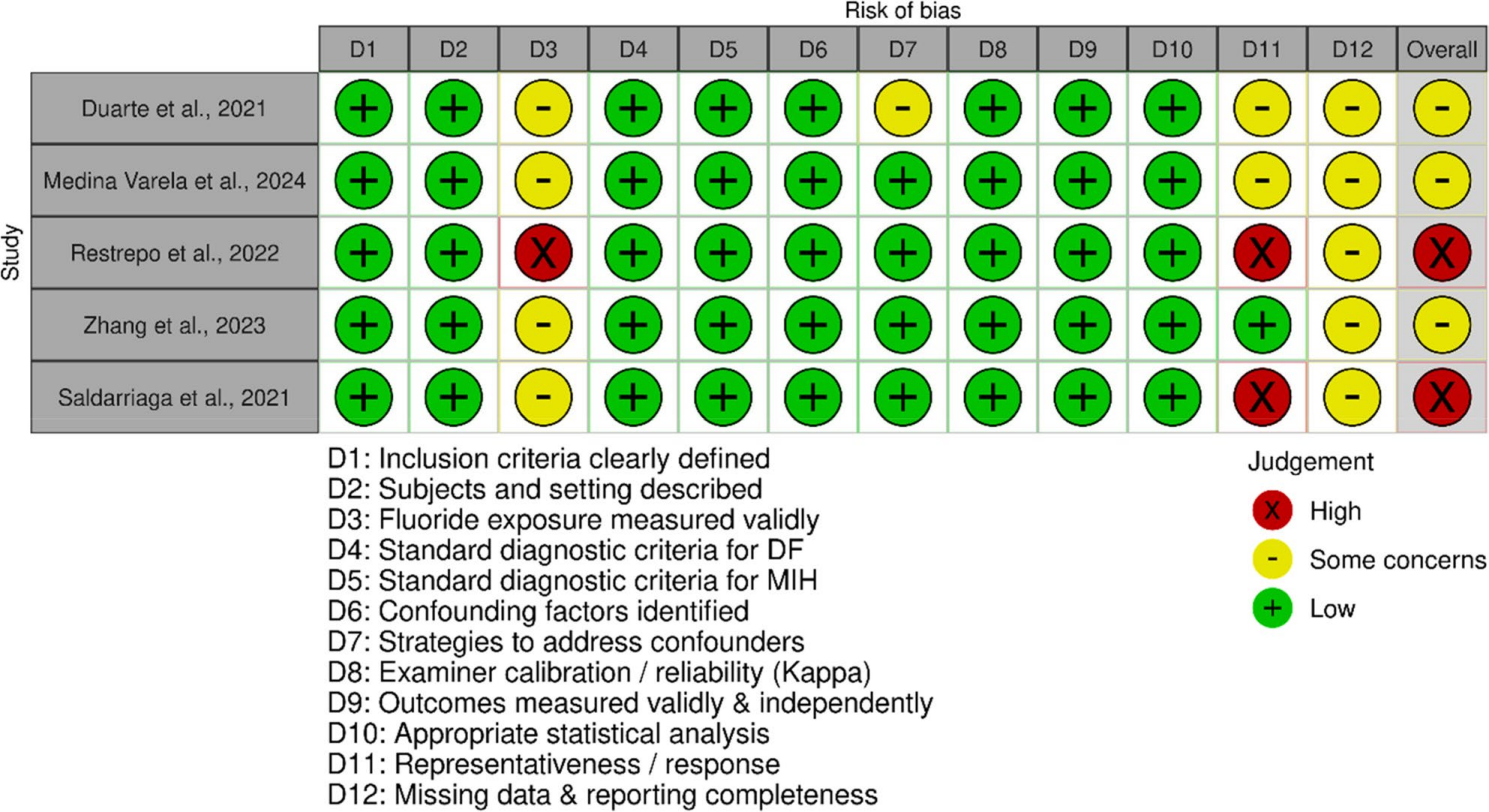
Risk of bias assessment. Risk of bias assessment of the included cross-sectional studies using the adapted 12-domain Joanna Briggs Institute (JBI) framework. Each domain was rated as low risk (+), some concerns (–), or high risk (×). The visualization was generated using the Robvis tool

The methodological quality of the five included studies was evaluated using the adapted 12-domain JBI-based framework. Overall, three studies were judged as having a moderate risk of bias, and two as having a high risk of bias. The main sources of concern were related to fluoride exposure assessment, as most studies relied on community-level indicators (water or salt fluoridation) rather than individual fluoride consumption, and to incomplete reporting of response rates or missing data. Nevertheless, all studies applied standardized diagnostic criteria for MIH and DF and reported examiner calibration supporting their internal validity. The results of this assessment are visualized using the Robvis tool in Fig. 2 (Fig. 2).

### Characteristics of the population with DF

The included studies were conducted in Colombia, Mexico, Brazil, and China. Three studies explicitly reported being conducted in fluoride-endemic areas, whereas two studies did not specify the endemic status. The total sample size from the five cross-sectional studies comprised 3,071 participants aged between 8 and 16 years, of whom 51.7% were females (Table 2). All studies reported a confirmed presence of DF within their populations, assessed primarily through the Thylstrup and Fejerskov Index (TFI). Examiner agreement for DF diagnosis was assessed using Cohen’s kappa (κ). Intra- and inter-examiner κ values ranged from 0.65 to 0.89 and from 0.67 to 0.92, respectively (Table 2). The prevalence of DF ranged from 25.5% to 98.7% (Table 3).

**Table 2:** Characteristics of the eligible studies

Authors	Country	Participants (*N*/Sex)	Age (years)	Training of examiners	κ DF	κ MIH
Saldarriaga et al., (2021) [18]	Colombia	77/ F = 39 M = 37	8–12	Yes	0.89*; 0.87**	NS
Medina Varela et al., (2024) [16]	Mexico	573/ F = 277 M = 296	8–12	Yes	0.89	0.87
Duarte et al., (2021) [19]	Brazil	400/ F = 229 M = 171	11–14	Yes	0.65*; 0.70*; 0.67**	0.83*; 0.78*; 0.88**
Zhang et al., (2023) [20]	China	1568/ F = 813 M = 755	8–10	Yes	0.89*; 0.92**	0.77*; 0.65**
Restrepo et al., (2022) [21]	Colombia	453/ F = 228 M = 225	13–16	NS	0.89	0.87

*N *Sample size, *DF *Dental Fluorosis, *MIH *Molar Incisor Hypomineralization, *NS *Not specified

*Intra-examiner kappa

**Inter-examiner kappa

**Table 3:** Prevalence of DF in Eligible Studies

Authors	Endemic area of DF	Fluoridated water	Fluoridated salt	Fluoride concentration (ppm or mg/L)	Index used for diagnosing DF	Prevalence of DF %
Saldarriaga et al., (2021) [18]	Yes	No	Yes	180–220** 0.1*	TFI	98.7
Medina Varela et al., (2024) [16]	Yes	Yes	NS	1.39*	TFI	70.9
Duarte et al., (2021) [19]	NS	NS	NS	NS	TFI	25.5
Zhang et al., (2023) [20]	Yes	Yes	NS	1.22–3.9*	TFI	55.0
Restrepo et al., (2022) [21]	NS	NS	Yes	180–220**	TFI	28.7

*NS *Not specified, *TFI *Thylstrup and Fejerskov Index

*Fluoride concentration in water

**Fluoride concentration in salt

### Prevalence, severity and co-occurrence of MIH in the population with DF

The prevalence of MIH ranged from 13.7% to 37.7% across the five included studies. Three studies utilized the diagnostic criteria established by the EAPD, one study applied the MIH Severity Scoring System (MIH-SSS), and the other used the index proposed by Ghanim et al. [11, 12]. Examiner agreement for MIH diagnosis was assessed using the κ index. Intra- and inter-examiner κ values ranged from 0.77 to 0.83 and from 0.65 to 0.88, respectively (Table 2). The proportion of individuals presenting both MIH and DF varied from 3.7% to 26%. Four studies reported MIH severity. Mild cases were the most prevalent (mean prevalence 55.1%), ranging from 42.6% to 68.8%, followed by moderate cases (mean prevalence 17.8%), varying between 0% and 37.9%, and severe cases (mean prevalence 27.1%), ranging from 6.4% to 41.7% (Table 4). A statistically significant association between MIH and DF was observed (χ² = 10.1, df = 1, *p* = 0.002; Fisher’s exact test: *p* = 0.002). The calculated OR was 1.36 (95% CI: 1.12–1.65).

**Table 4:** Prevalence of MIH and its Association with DF

Authors	Number of teeth affected by MIH	Index used for diagnosing MIH	Prevalence of MIH %	Prevalence of MIH in patients with DF %	Severity of MIH %
Saldarriaga et al., (2021) [18]	NS	EAPD	14.4	14.4	NR
Medina Varela et al., (2024) [16]	NS	EAPD	37.7	26.0	Mild: 42.6 Moderate: 37.9 Severe: 19.5
Duarte et al., (2021) [19]	227	MIH-SSS	18.0	4.0	Mild: 50.0 Moderate: 8.3 Severe: 41.7
Zhang et al., (2023) [20]	482	EAPD	13.7	3.7	Mild: 59.1 Severe: 40.9
Restrepo et al., (2022) [21]	231	Ghanim et al., (2015) [11] Ghanim et al., (2019) [12]	31.0	11.5	Mild: 68.8 Moderate: 24.8 Severe: 6.4

*DF *Dental Fluorosis, *MIH *Molar Incisor Hypomineralization, *NR *Not reported, *NS *Not specified, *MIH-SSS *MIH Severity Scoring System, *EADP *European Academy of Pediatric Dentistry

### Meta-analysis of the association between MIH and DF

**Fig. 3:**
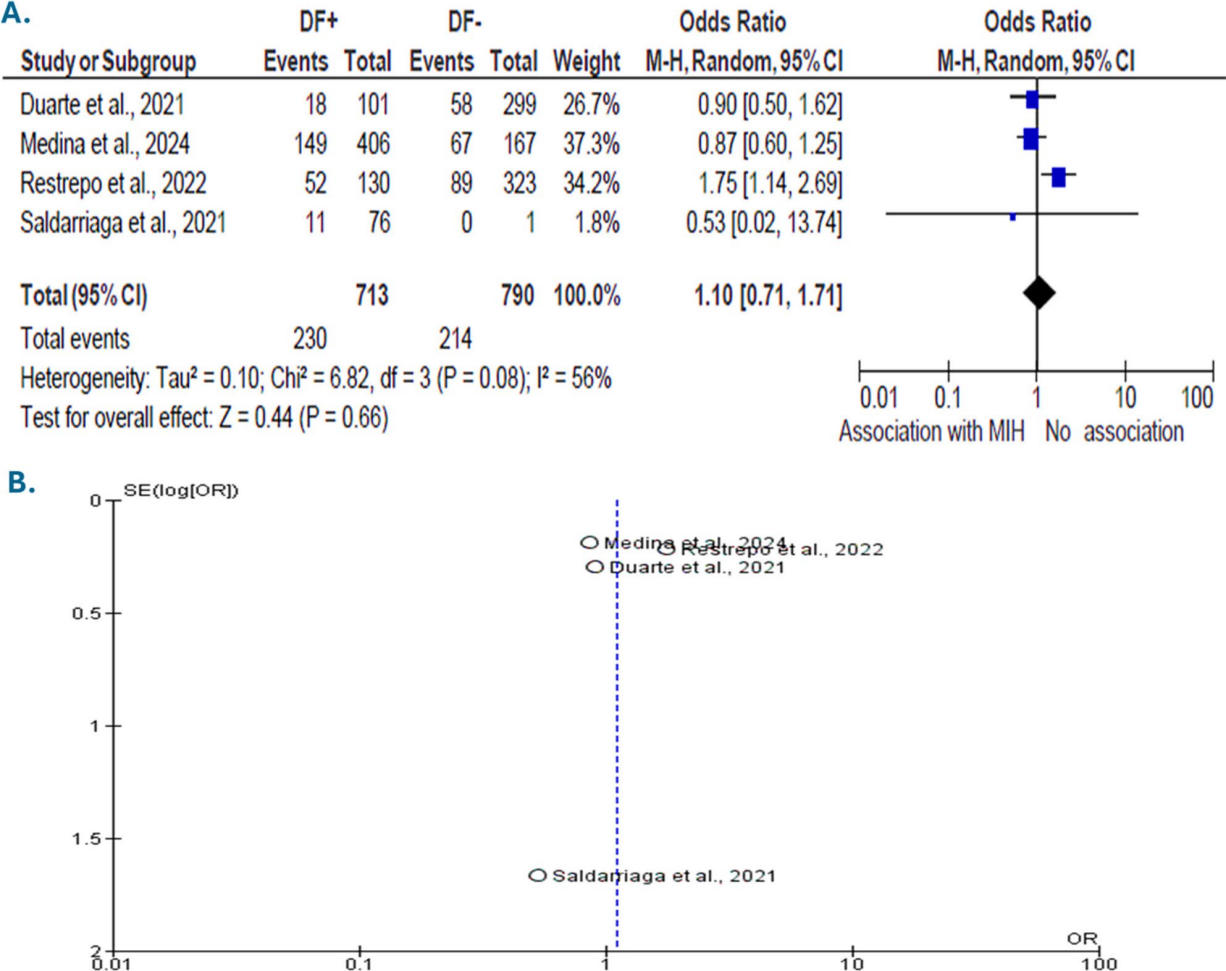
Forest and funnel plots for the association between MIH and DF. **A** Forest plot showing the pooled OR and 95% CIs from the random-effects meta-analysis assessing the association between MIH and DF. The summary diamond overlaps the line of no effect (OR = 1), indicating no significant association. **B** Funnel plot illustrating the assessment of potential publication bias among the included studies on the MIH–DF association. No apparent asymmetry was observed, although the limited number of studies (k = 4) restricts formal evaluation

All five included studies reported MIH prevalence. However, a pooled prevalence of MIH among children with DF was not performed because of substantial heterogeneity across prevalence estimates. In contrast, four studies provided extractable 2 × 2 data. They were included in the meta-analysis assessing the association between MIH and DF. The total sample included 1,503 children; 713 had DF, and 790 did not. One study was excluded as it used a machine-learning Thylstrup and Fejerskov Index approach and did not provide the raw cross-tabulated data required for pooling. Between-study heterogeneity was moderate (χ² = 6.82; df = 3; *p* = 0.08; I² = 56%; τ² = 0.10), supporting the use of a random-effects model. Using a random-effects Mantel–Haenszel model, the pooled OR for MIH in children with DF compared with those without DF was 1.10 (95% CI 0.71–1.71; Z = 0.44, *p* = 0.66). The summary diamond on the forest plot overlapped the line of no effect (OR = 1) **(**Fig. 3.A). The funnel plot **(**Fig. 3.B) showed no apparent asymmetry. Given the limited number of studies (*k = 4*), the assessment of publication bias remains inconclusive.

## Discussion

To our knowledge, this is the first systematic review quantifying the prevalence and co-occurrence of MIH among children diagnosed with DF. Our study was based on five cross-sectional studies from Colombia, Mexico, Brazil, and China, which collectively included 3,071 participants aged 8–16 years. From 287 records initially identified across PubMed, Web of Science, and Scopus, only five studies met all inclusion criteria after full-text screening. The limited number of eligible studies reflects the current scarcity of epidemiological data simultaneously assessing the prevalence of MIH and DF within the same population. In several potentially relevant studies, the prevalence of one of the two conditions was not available or not reported, leading to their exclusion. All included studies were cross-sectional in design, which was justified because no longitudinal or case–control studies have yet explored the co-occurrence of MIH and DF. Cross-sectional designs are the most appropriate for estimating prevalence and providing an initial overview when higher-level analytical evidence is lacking. The concentration of available evidence in only four countries, Colombia, Mexico, Brazil, and China (Table 2), illustrates a geographic imbalance in global research output, thereby limiting the external validity and generalizability of findings. These selection characteristics emphasize that the small number of retained studies does not reflect methodological shortcomings of this review but rather a knowledge gap in the current literature, highlighting the need for further epidemiological investigations across populations in which dental enamel is altered to different fluoride levels.

The risk-of-bias assessment (Fig. 2) showed that three studies had a moderate risk of bias and two a high risk. The main concerns were the indirect assessment of fluoride exposure, often based on community rather than individual data, and incomplete reporting of response rates or missing data. Nevertheless, all studies used standardized diagnostic criteria for MIH and DF, and reported satisfactory examiner calibration (κ ≥ 0.65) (Table 2), supporting internal validity. The overall evidence from the included studies can be considered of moderate quality, with heterogeneity likely linked to methodological differences rather than consistent epidemiological patterns.

Our results revealed that the prevalence of DF ranged from 25.5% to 98.7%, reflecting regional differences in fluoride exposure. Among the three studies involving fluoride-endemic areas [16, 18, 20], the main source of fluoride was drinking water and salt. Drinking water is significantly associated with increased DF risk among children and adolescents [8]. Notably, not only the risk of DF but also its severity is linked to the consumption of fluoride in drinking water [9]. All the studies included in this review used the TFI to evaluate the severity of DF and showed different scores. However, none of the included studies reported a direct correlation between the fluoride concentration in water or salt and the severity of DF. In the literature, a significant correlation between fluoride levels in drinking water and the severity of DF has been demonstrated in Indian communities, with individuals exposed to > 1.5 ppm fluoride in drinking water being 1.8 times more likely to develop moderate to severe fluorosis [9]. To obtain comparable data, studies should not only use the same index to evaluate DF but also analyze the fluoride in drinking water.

Our study revealed that the prevalence of MIH ranged from 14.4% to 37.7%, depending on the diagnostic criteria applied and the geographic location of the studies (China, Brazil, Mexico, and Colombia). When integrating findings from two large meta-analyses and one umbrella review, the overall global burden of MIH consistently falls between 12% and 15%, with substantial geographical variability across regions [24–26]. To classify MIH and its severity, the included studies used either the criteria of the EAPD [27, 28], the MIH-SSS [29], or the criteria developed by Ghanim et al. (2015, 2019) [11, 12]. Interestingly, the prevalence of MIH seems to vary depending on the diagnostic criteria applied. In fact, an umbrella review [26] and meta-analysis [24] reported a higher mean prevalence of 14.5% in clinical studies when the EAPD classification was employed, compared with 10.2% when alternative definitions were used [30]. The severity of MIH has been reported in most studies, with mild forms predominating overall. Moreover, our findings indicated notable geographic variability, which is consistent with global epidemiological patterns. From a clinical standpoint, distinguishing MIH from DF remains particularly challenging, as both conditions may present as diffuse or demarcated opacities with overlapping color patterns. Diagnostic difficulties are well documented in the literature, with studies showing that even trained clinicians may struggle to differentiate between developmental enamel defects with similar visual presentations, including MIH, DF, and other enamel opacities [31]. Mild MIH lesions can mimic the chalky white or yellow-brown opacities typical of fluorosis, leading to possible misclassification, especially in epidemiological studies lacking standardized calibration [23]. However, despite these visual similarities, MIH and DF differ in key clinical characteristics, including lesion demarcation, distribution, and the teeth typically affected. MIH presents as asymmetrical, well-demarcated defects, commonly associated with hypersensitivity and primarily affecting the FPMs. In contrast, DF shows diffuse, symmetrical involvement of multiple teeth, with poorly demarcated opacities. In addition, DF mainly affects the superficial enamel layer, whereas MIH may also involve deeper enamel near the enamel–dentin junction, suggesting a different treatment approach. These observations highlight the importance of adopting standardized diagnostic and severity criteria in future MIH research to minimize diagnostic overlap and improve data comparability.

Concerning the co-occurrence and the association between MIH and DF, the descriptive synthesis of the included studies initially suggested a significant association (χ² = 10.1, df = 1, *p* = 0.002; OR = 1.36, 95% CI: 1.12–1.65). However, when accounting for inter-study variance through a random-effects meta-analysis, this relationship was not statistically confirmed (OR = 1.10, 95% CI: 0.71–1.71; Z = 0.44, *p* = 0.66), with moderate heterogeneity (I² = 56%). This discrepancy highlights that aggregated descriptive data can overestimate associations when methodological heterogeneity is not considered. The forest plot (Fig. 3.A) shows that the pooled estimate overlaps the line of no effect, indicating no consistent trend, while the funnel plot (Fig. 3.B) reveals no apparent publication bias, although the limited number of studies (k = 4) restricts formal testing. Two hypotheses can be drawn from these results. First, MIH and DF may represent distinct enamel disturbances with independent etiopathogenic pathways. Alternatively, methodological variability, particularly in fluoride exposure assessment and diagnostic overlap, could mask an underlying relationship. Some studies have reported a protective association of high-severity fluorosis (TFI ≥ 4) with reduced MIH severity [16, 20], whereas others have suggested a possible aggravating role of fluoride depending on concentration and exposure timing [17]. The severity of MIH in our dataset further supports this complexity, with mild cases predominating (mean 55.1%). An in vivo study using a rat model further supported the potential interaction between fluoride exposure and enamel hypomineralization [22]. Rats exposed to NaF alone, compared to those exposed to NaF bisphenol A, chemical compounds of various plastics, developed more severe enamel defects, with significant changes in the expression of amelogenesis-related genes such as Amelx, Enam, Klk4, and Slc26a4. These data confirm that fluoride may contribute to enamel defects when it acts in synergy with other environmental disruptors [22].

From a practical perspective, these findings also hold clinical relevance for pediatric dentists. In fact, recognizing the potential diagnostic overlap between MIH and DF is crucial for accurate diagnosis and appropriate decision-making. Correct differentiation allows clinicians to tailor preventive and restorative strategies according to the lesion’s etiology and severity, while fluorosis management primarily focuses on aesthetic correction, MIH often requires desensitizing care and early intervention.

### Limitations

This systematic review and meta-analysis present several limitations that should be acknowledged. Firstly, only five cross-sectional studies met the inclusion criteria, and four provided extractable data for meta-analysis, resulting in a relatively small sample (*n* = 1,503). In addition, the overall methodological quality of the included studies was moderate to low, as reflected by the risk-of-bias assessment. This reflects the current scarcity of epidemiological data simultaneously assessing MIH and DF in the literature. The exclusive inclusion of cross-sectional designs, although suitable for estimating prevalence, limits causal inference.

Secondly, considerable heterogeneity among studies was observed due to differences in diagnostic criteria, fluoride exposure assessment, and population profiles. The absence of a standardized index capable of jointly evaluating MIH and DF may have contributed to diagnostic misclassification. Moreover, developing a combined diagnostic index for MIH and DF would first require stronger evidence confirming whether these two conditions share common etiological pathways or not. Additionally, since none of the included studies directly quantified individual fluoride intake; exposure was estimated indirectly using environmental indicators, introducing some uncertainty in exposure assessment.

Finally, the limited geographic diversity, focused on studies from Colombia, Mexico, Brazil, and China, restricts the generalizability of findings to other populations.

Despite these constraints, this review provides the first quantitative synthesis exploring the MIH-DF relationship and underscores the need for standardized diagnostic tools, improved exposure assessment, and multicenter longitudinal studies to better understand their epidemiological pattern.

## Conclusion

This systematic review and meta-analysis provides a quantitative synthesis of the available evidence on the prevalence of MIH among children with DF. Although descriptive analyses suggested a possible association, the meta-analytic findings did not demonstrate a consistent relationship between the two conditions. The available evidence reveals substantial methodological heterogeneity, particularly regarding fluoride exposure assessment and diagnostic approaches. Further well-designed, population-based studies using standardized diagnostic criteria and improved exposure assessment are needed to better characterize the epidemiology of DDEs and their potential co-occurrence. This research also contributes to the United Nations Sustainable Development Goals by promoting a better understanding of environmental influences on oral health.

## Supplementary Information

Supplementary Material 1.

## Data Availability

All data analyzed during this study are included in this published article and its supplementary information files.

## Abbreviations

CI: Confidence interval
df: Degrees of freedom
DF: Dental fluorosis
DDE(s): Developmental defect(s) of enamel
FPM(s): First Permanent Molars
EAPD: European Academy of Pediatric Dentistry
I²: Inconsistency statistic (between-study heterogeneity)
JBI: Joanna Briggs Institute
κ: Cohen’s kappa (inter-/intra-examiner agreement)
MIH: Molar Incisor Hypomineralization
MIH-SSS: MIH Severity Scoring System
MIH-TNI: MIH Treatment Need Index
NaF: Sodium fluoride
OR: Odds ratio
PECOS: Population, Exposure, Comparator, Outcome, Study design
ppm: Parts per million
PRISMA: Preferred Reporting Items for Systematic Reviews and Meta-Analyses
PROSPERO: International Prospective Register of Systematic Reviews
RevMan: Review Manager
TFI: Thylstrup and Fejerskov Index
TSIF: Tooth Surface Index of Fluorosis
τ²: Between-study variance (random-effects model)
χ²: Chi-square statistic
