# Supplementary material for: Prevalence and association of Molar Incisor Hypomineralization (MIH) in patients presenting dental fluorosis: a systematic review and meta-analysis
Source: BMC Oral Health. 2026 Mar 7;26:929. doi: 10.1186/s12903-026-08052-9 (PMC13224572; doi:10.1186/s12903-026-08052-9)
Supplement: Supplementary file 1 [file 12903_2026_8052_MOESM1_ESM.odt]

Table1: Search string for Pubmed database search 

| Tag | Subject Search | Search String |  | Pubmed synonyms | Number of articles |
| --- | --- | --- | --- | --- | --- |
| #1 | mih | ("Molar Hypomineralization"[Mesh]) OR “MIH” | Hypomineralization, Molar Molar Hypomineralizations Molar Incisor Hypomineralization Hypomineralization, Molar Incisor Molar Incisor Hypomineralizations Incisor Molar Hypomineralization Hypomineralization, Incisor Molar Incisor Molar Hypomineralizations Molar Hypomineralization, Incisor Incisor Hypomineralization Hypomineralization, Incisor Incisor Hypomineralizations MIH | 1,637 |
| #2 | Dental fluorosis | ("Fluorosis, Dental"[Mesh]) OR "Fluorides"[Mesh] | Dental Fluorosis Dental Fluoroses Fluoroses, Dental Mottled Teeth Teeth, Mottled Mottled Enamel Enamel, Mottled Mottled Enamels Fluoride | 42,092 |
| #3 | #1 AND #2 AND | (#1) AND (#2)  (("Molar Hypomineralization"[Mesh]) OR "MIH") AND (("Fluorosis, Dental"[Mesh]) OR "Fluorides"[Mesh]) |  | 52 |

Table 2: Search string for Scopus database search 

| Tag | Subject Search | Search String | Number of articles |
| --- | --- | --- | --- |
| #1 | MIH | ("Molar Hypomineralization") OR (“Hypomineralization Molar”) OR (“Molar Hypomineralizations”) OR (“Molar Incisor Hypomineralization”) OR (“Hypomineralization, Molar Incisor”) OR (“Molar Incisor Hypomineralizations”) OR (“Incisor Molar Hypomineralization”) OR (“Hypomineralization Incisor Molar”) OR (“Incisor Molar Hypomineralizations”) OR (“Molar Hypomineralization Incisor”) OR (“Incisor Hypomineralization”) OR (“Hypomineralization, Incisor”) OR (“Incisor Hypomineralizations”) OR (“MIH”) | 2493 |
| #2 | Dental fluorosis | ("Fluorosis Dental") OR (“Dental Fluorosis”) OR (“Dental Fluoroses”) OR (“Fluoroses Dental”) OR (“Mottled Teeth”) OR (“Teeth Mottled”) OR (“Mottled Enamel “) OR (“Enamel Mottled “) OR (“Mottled Enamels”) OR (“Fluorides”) OR (“Fluoride”) | 204,411 |
| #3 | #1 AND #2 AND | (#1) AND (#2) ("Molar Hypomineralization") OR (“Hypomineralization Molar”) OR (“Molar Hypomineralizations”) OR (“Molar Incisor Hypomineralization”) OR (“Hypomineralization, Molar Incisor”) OR (“Molar Incisor Hypomineralizations”) OR (“Incisor Molar Hypomineralization”) OR (“Hypomineralization Incisor Molar”) OR (“Incisor Molar Hypomineralizations”) OR (“Molar Hypomineralization Incisor”) OR (“Incisor Hypomineralization”) OR (“Hypomineralization, Incisor”) OR (“Incisor Hypomineralizations”) OR (“MIH”) AND ("Fluorosis Dental") OR (Fluoride) OR (“Dental Fluorosis”) OR (“Dental Fluoroses”) OR (“Fluoroses Dental”) OR (“Mottled Teeth”) OR (“Teeth Mottled”) OR (“Mottled Enamel “) OR (“Enamel Mottled “) OR (“Mottled Enamels”) OR (“Fluorides”) | 125 |

Table 3: Search string for Web of science database search 

| Tag | Subject Search | Search String | Number of articles |
| --- | --- | --- | --- |
| #1 | MIH | ("Molar Hypomineralization") OR (“Hypomineralization Molar”) OR (“Molar Hypomineralizations”) OR (“Molar Incisor Hypomineralization”) OR (“Hypomineralization, Molar Incisor”) OR (“Molar Incisor Hypomineralizations”) OR (“Incisor Molar Hypomineralization”) OR (“Hypomineralization Incisor Molar”) OR (“Incisor Molar Hypomineralizations”) OR (“Molar Hypomineralization Incisor”) OR (“Incisor Hypomineralization”) OR (“Hypomineralization, Incisor”) OR (“Incisor Hypomineralizations”) OR (“MIH”) |  |
| #2 | Dental fluorosis | ("Fluorosis Dental") OR (“Dental Fluorosis”) OR (“Dental Fluoroses”) OR (“Fluoroses Dental”) OR (“Mottled Teeth”) OR (“Teeth Mottled”) OR (“Mottled Enamel “) OR (“Enamel Mottled “) OR (“Mottled Enamels”) OR (“Fluorides”) OR (“Fluoride”) |  |
| #3 | #1 AND #2 AND | (#1) AND (#2) ("Molar Hypomineralization") OR (“Hypomineralization Molar”) OR (“Molar Hypomineralizations”) OR (“Molar Incisor Hypomineralization”) OR (“Hypomineralization, Molar Incisor”) OR (“Molar Incisor Hypomineralizations”) OR (“Incisor Molar Hypomineralization”) OR (“Hypomineralization Incisor Molar”) OR (“Incisor Molar Hypomineralizations”) OR (“Molar Hypomineralization Incisor”) OR (“Incisor Hypomineralization”) OR (“Hypomineralization, Incisor”) OR (“Incisor Hypomineralizations”) OR (“MIH”) AND ("Fluorosis Dental") OR (Fluoride) OR (“Dental Fluorosis”) OR (“Dental Fluoroses”) OR (“Fluoroses Dental”) OR (“Mottled Teeth”) OR (“Teeth Mottled”) OR (“Mottled Enamel “) OR (“Enamel Mottled “) OR (“Mottled Enamels”) OR (“Fluorides”) | 104 |
